# Significant variability in surgeons’ preferred correction maneuvers and instrumentation strategies when planning adolescent idiopathic scoliosis surgery

**DOI:** 10.1186/s13013-018-0169-8

**Published:** 2018-10-07

**Authors:** Franck Le Navéaux, A. Noelle Larson, Hubert Labelle, Carl-Eric Aubin, Minimize Implants Maximize Outcomes Study Group

**Affiliations:** 1 Department of Mechanical Engineering, Polytechnique Montreal, P.O. Box 6079, Downtown Station, Montreal, QC H3C 3A7 Canada; 2 Research Center, Sainte-Justine University Hospital Center, 3175, Cote Sainte-Catherine Road, Montreal, QC H3T 1C5 Canada; 3 Mayo Clinic, 200 1st Street SW, Rochester, MN 55902 USA; 4 Canada Research Chair in Orthopedic Engineering, and NSERC/Medtronic Industrial Research Chair in Spine Biomechanics, Department of Mechanical Engineering, Polytechnique Montreal, P.O. Box 6079, Downtown Station, Montreal, QC H3C 3A7 Canada

**Keywords:** Adolescent idiopathic scoliosis, Implant density, Implant pattern, Surgical planning, Correction maneuvers

## Abstract

**Background:**

Increased implant number is thought to provide better control on the scoliotic spine, but there is limited scientific evidence of improved deformity correction and surgical outcomes with high-density constructs. The objective is to assess key anchor points used by experienced spinal deformity surgeons and to evaluate the effect of implant density pattern on correction techniques.

**Methods:**

Seventeen experienced spine surgeons reviewed five Lenke 1 adolescent idiopathic scoliosis cases and provided their preferred posterior correction technique (implant pattern, correction maneuvers, and implants used for their execution) and an alternative technique with the minimal implant density they felt would be acceptable (170 surgical plans total). Additionally, for each case, they selected acceptable screw patterns for surgery from seven published implant configurations. Variability in the surgeons’ plans was assessed, including instrumentation and correction strategies.

**Results:**

The preferred correction plan involved an average of 1.65 implants/vertebra, with 88% of the available anchor points at the apex ± 1 vertebra used for the execution of correction maneuvers and only 43% of possible anchor points used proximal and distal to the apical area. The minimal density that surgeons found acceptable was 1.24 implants/vertebra. The minimal density plan involved more in situ rod contouring (53 vs. 41%), fewer vertebral derotation maneuvers (82 vs. 96%), and fewer implants used for compression/distraction maneuvers (1.18 and 1.42 respectively) (*p* < 0.05). Implant placement at alternate levels or dropout of convex implants above and below the apical area was most frequently considered acceptable (> 70% agreement).

**Conclusions:**

Implant position and number affect surgeons correction maneuvers selection. For low implant density constructs, dropout in the convexity and particularly in the periapical region is accepted by surgeons, with minor influence on planned correction maneuvers. Thus, preoperative implant planning must take into account which anchor points are needed for desired correction maneuvers.

## Highlights

- The surgeons varied their correction techniques based on implant density.

- Contemporary maneuvers justify the use of high implant density in the apical region compared to other areas of the construct.

- Most surgeons considered it acceptable to alternate and/or skip implants in the convexity, particularly in the regions above and below the apex.

## Background

Pedicle screw instrumentation is now widely used and has led to improved correction techniques for the treatment of complex spinal deformities compared to hook only or hybrid constructs [1–3]. As surgeons have become more familiar with insertion techniques, many surgeons use higher implant density constructs, or more screws per level fused [4]. High pedicle screw density is thought to allow for enhanced capacity to perform sophisticated maneuvers to correct 3D spinal deformities [5]. However, there is limited evidence that high-density pedicle screw constructs result in improved deformity correction and surgical outcomes [6, 7]. Moreover, a high-density construct has potential drawbacks such as exaggerated lordosis, increased surgical time, blood loss, radiation exposure, and expense [8–12]. Given these considerations, constructs with fewer pedicle screws, if proven safe and clinically equivalent, would improve surgical outcomes and value.

Among experienced spine surgeons, there is a significant variability of fused levels [13], as well as significant variability of acceptable implant density and patterns, reflecting a lack of consensus about optimal screw density [14]. Several patterns with varying anchor distributions over the fused segment have been proposed in the literature as alternatives to fully instrumented constructs [7, 15–19]. However, the number and location of anchor points dictate which correction maneuvers can be used, and not all implant patterns are readily compatible with contemporary correction techniques. With optimized correction maneuvers linked to the specific screw pattern, it may be possible to obtain adequate correction results using fewer screws. Thus, identification of key anchor points necessary for specific corrective maneuvers could be the basis for rational implant pattern design and the justification for possible screw dropout. Unfortunately, little work has been completed on this topic to date.

The purpose of this study was to assess the anchor points used by experienced spinal deformity surgeons to execute each specific correction maneuver. Further, we sought to determine how the surgeon’s technique to deformity correction changed after switching to a low implant density construct.

## Methods

### Study population

**Fig. 1:**
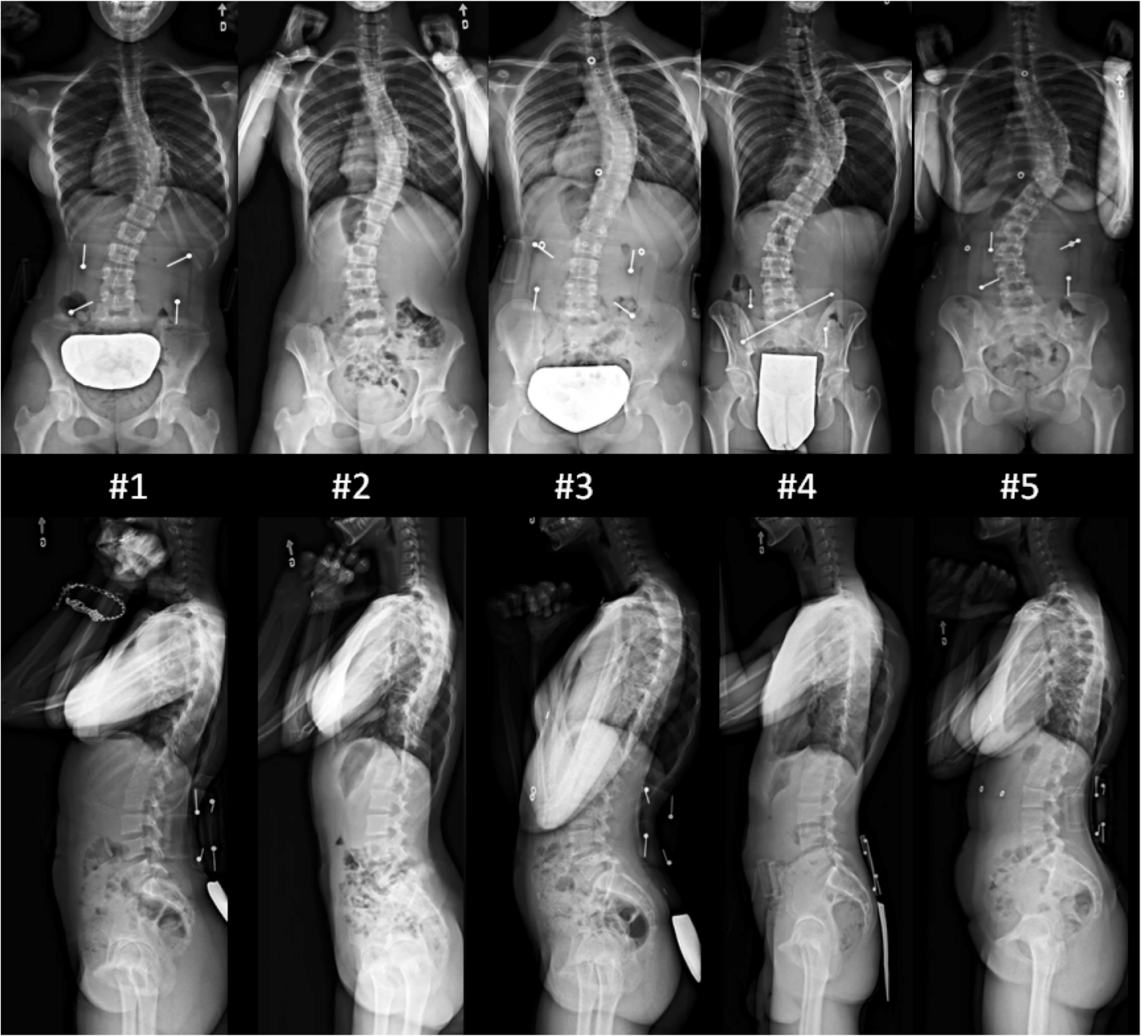
Preoperative standing posteroanterior and lateral radiographs of the five cases

Seventeen experienced spine surgeons practicing in North America agreed to participate in this study. Mean years of experience for the surgeons was 21 (range, 5–40 years). All surgeons were given the de-identified cases in a similar fashion. Institutional review board approval was not required for this study as each of the subjects (surgeons) participated as part of a quality study. Each subject (surgeon) independently reviewed and completed detailed surgical plans for five Lenke 1 adolescent idiopathic scoliosis (AIS) cases of patients who were candidates for posterior surgical fusion. Cases were selected that represented a variety of curve magnitudes and stiffness for a primary main thoracic right curve pattern, which is the most common spinal deformity in AIS [20]. Three patients with Lenke 1A, one with Lenke 1B, and one with Lenke 1C were selected (Fig. 1). Main thoracic curve Cobb angle ranged from 48 to 66° (Table 1). Preoperative curve flexibility assessed on bending films ranged from 27 to 73%. Thoracic kyphosis (T5-T12) ranged from 9 to 25° and apical vertebral rotation from 16 to 23°.

**Table 1:** Demographic data and preoperative curve characteristics of patients

Patient numbers	#1	#2	#3	#4	#5
Age at surgery (years)	13	14	18	18	18
Lenke type	1A	1A	1A	1B	1C
Proximal thoracic Cobb angle (°)	38	45	34	39	49
Main thoracic (MT) Cobb angle (°)	54	47	48	66	66
Lumbar Cobb angle (°)	34	35	22	50	56
MT curve flexibility (% reduction)	54	73	60	28	44
Thoracic kyphosis (T5-T12) (°)	19	15	23	9	25
Lumbar lordosis (T12-S1) (°)	48	41	58	51	44
Apical vertebral rotation (°)	19	16	23	22	19
Selected fusion levels	T4-L1	T4-T12	T4-L1	T2-L2	T3-L1

### Survey

For each case, surgeons were provided with preoperative standing posteroanterior, lateral, and supine side bending radiographs with Cobb angle measurements reported for each curve. The survey was divided into three parts:

Part 1

**Fig. 2:**
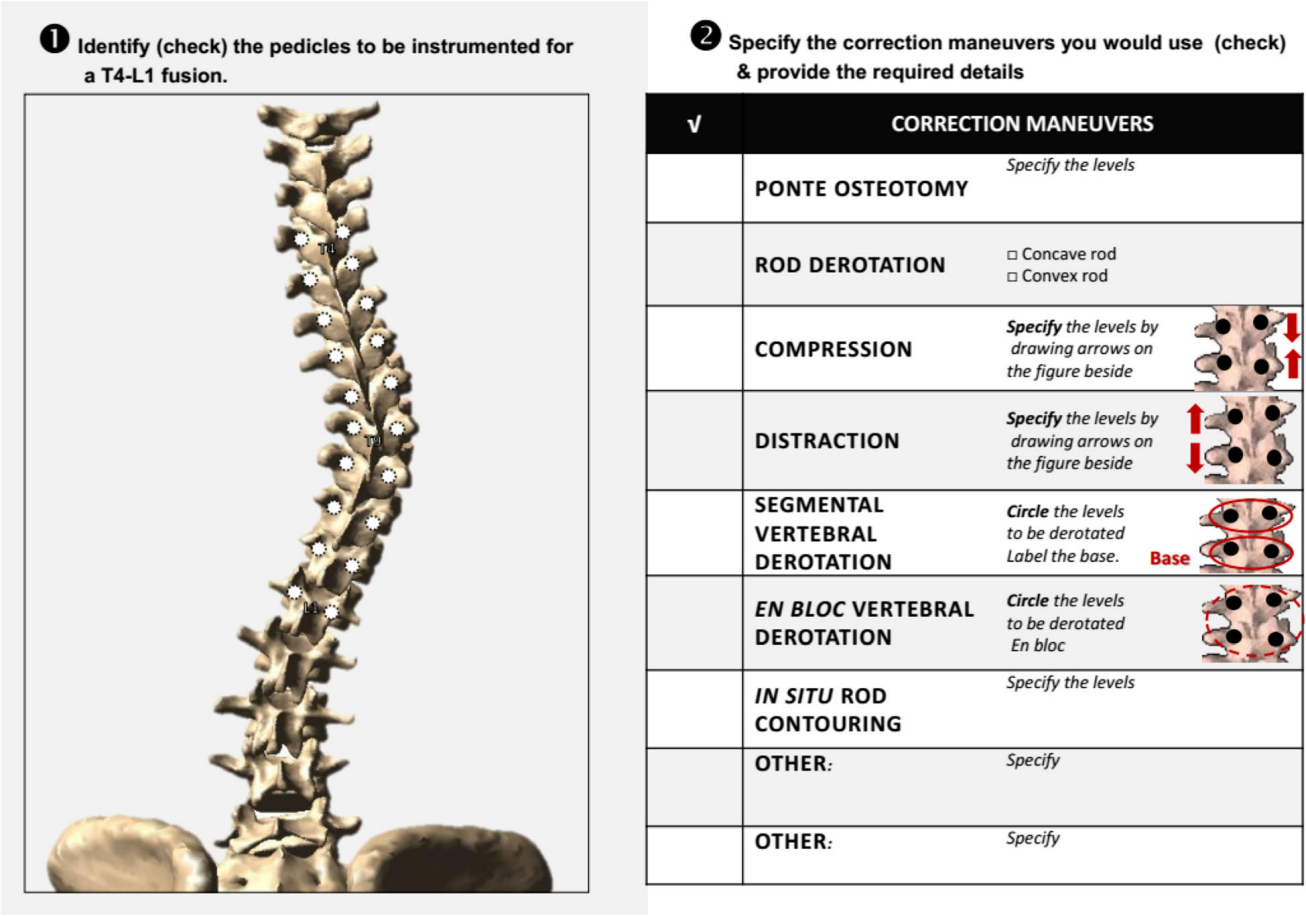
Worksheet used to identify implant distribution and correction maneuvers planned for the treatment of each patient (here, case 1)

Using worksheets with posteroanterior projections of 3D reconstructed models of the patients’ spine with predetermined fusion levels (Fig. 2), surgeons were required to provide their operative plan including implant placement. They were then asked to select the correction maneuvers they would perform from a list of common techniques used for the treatment of AIS: Ponte osteotomy, rod derotation, compression, distraction, segmental vertebral derotation, en bloc vertebral derotation, and in situ contouring. They also had the possibility to specify any other correction maneuver they would use. For each specific maneuver, they had to identify the vertebral level and anchor points used.

Part 2

Surgeons were then asked to repeat the operative planning survey on an identical worksheet using the minimum number of implants that they would find acceptable for treatment.

Part 3

Surgeons were asked to select implant patterns they would find acceptable for surgical instrumentation for each case from a list of seven implant patterns reported in the literature. The implant patterns consisted of an *apical key vertebra* construct with both the two proximal and distal vertebrae instrumented as well as the convex pedicle of the apical vertebra and the two concave pedicles of the periapical vertebrae [7, 17, 18]; a *convex minimal* construct with the concave side fully instrumented and only four implants in the convexity on the two proximal and the two distal vertebrae [19]; *periapical dropout* constructs with vertebra other than the two proximal, two distal, and three apical left non-instrumented either on both sides or on convex side only [15]; an *alternate* construct with implant on every other vertebra on both sides or on the convex side only [7, 16]; and a *fully instrumented* construct with screw placed bilaterally on every segment.

### Data analysis

The 170 surgical plans were reviewed and analyzed to build a detailed implant map, including which implants were involved in the execution of each maneuver. In order to facilitate comparison and consistency between cases, we defined a standardized nomenclature to assess the distribution of screws across the levels fused and the location of specific maneuver execution. The implant construct was divided into five regions based on the apex of the curve and the extent of the fusion. The proximal region was defined as the upper instrumented vertebra (UIV) and one level caudad, and the distal region was defined as the lower instrumented vertebrae (LIV) and one level cephalad. The apical region includes the apical vertebra and its two adjacent vertebrae. Finally, two other regions composed of remaining vertebrae were established as the upper and lower periapical regions. The percentage of times that each instrumentation pattern was considered acceptable by the 17 surgeons was computed.

### Statistical analysis

All data were analyzed using STATISTICA V10 computer software (StatSoft Inc., Tulsa, OK, USA). The difference of densities used between cases and surgeons was evaluated with an analysis of variance (ANOVA one-way). A non-parametric Wilcoxon matched pair test was performed to evaluate differences in implant density between sides of the curve and between regions of the constructs. A McNemar test was used to evaluate changes in correction technique between surgeons’ preferred planning and the alternate planning with a lower implant density construct. Fleiss’ kappa was used to assess the reliability of agreement among surgeons about which implant patterns were acceptable for treatment. A power analysis was performed. The sample size of required operative plans was calculated taking into consideration 10% change in maneuver execution proportion with a confidence level of 95% and was set to ≥ 150 plans (15 surgeons × 5 patients × 2 operative plans). A *p* value less than 0.05 was considered statistically significant.

## Results

**Fig. 3:**
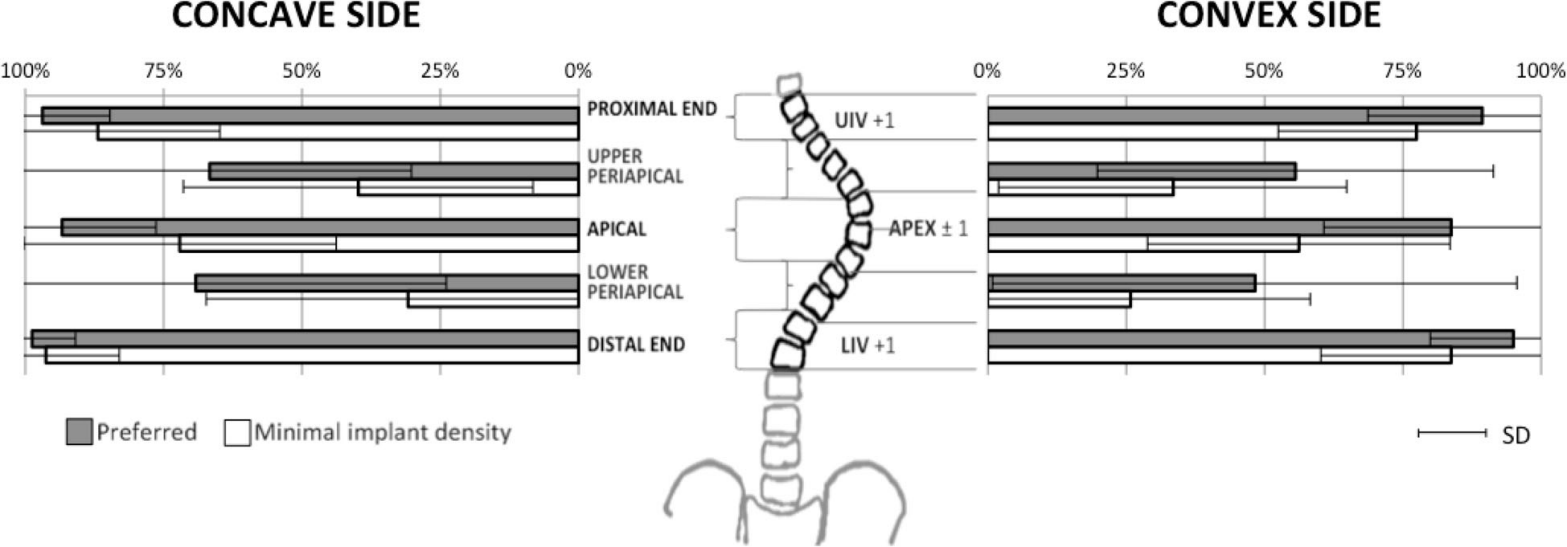
Percentage of available pedicles filled on five regions for the concave and convex sides of the instrumented spine for the preferred and minimal implant density techniques

Surgeons completed two plans for each of the five cases. Thus, a total of 170 surgical plans were developed, 85 using the surgeon’s preferred density and 85 using minimal acceptable implant density. The surgeons’ preferred operative plans used an average of 1.65 implants per vertebra (SD 0.25, range 1 to 2) compared to 1.24 (SD 0.21, range 0.85 to 1.82) for the minimal acceptable implant density constructs. Implant density selection was similar for the five cases (*p* > 0.05) but varied significantly among the surgeons (*p* < 0.01 for both scenarios). The surgeon with the highest density construct selected an average of 2 implants per vertebra for the preferred correction technique and 1.73 for the minimal density technique. In contrast, the surgeon using the fewest number of implants selected an average of 1.31 implants per vertebra for the preferred correction technique and 1.17 for the alternative technique. The surgeon with the greatest acceptable range of implant density selected an average of 2 implants per vertebra for the preferred correction technique and 1.05 for the alternative technique. Implant distribution was reported as the mean percentage of pedicles filled at each region of the instrumented spine for both the concave and convex sides of the curvature (Fig. 3). Surgeons’ operative plans involved a greater implant density on the concave side with a mean of 88% of available pedicles filled (SD 13%, range 54 to 100%) in contrast to a 77% mean (SD 15%, range 46 to 100%) on the convex side (*p* < 0.01). For the minimal implant density techniques, a mean of 67% (SD 15%, 46 to 100%) available pedicles were filled on the concave side and a mean of 58% (SD 9%, 38 to 82%) on the convex side (*p* < 0.01). Screw dropout between the two techniques was most commonly found in the concave lower periapical region with a mean decrease of 38% of pedicle filled (*p* < 0.01). Screw dropout was similar in the other apical and periapical regions (*p* > 0.05) with on average 24% fewer pedicles filled. Screw dropout was less common in the proximal and distal regions.

**Fig. 4:**
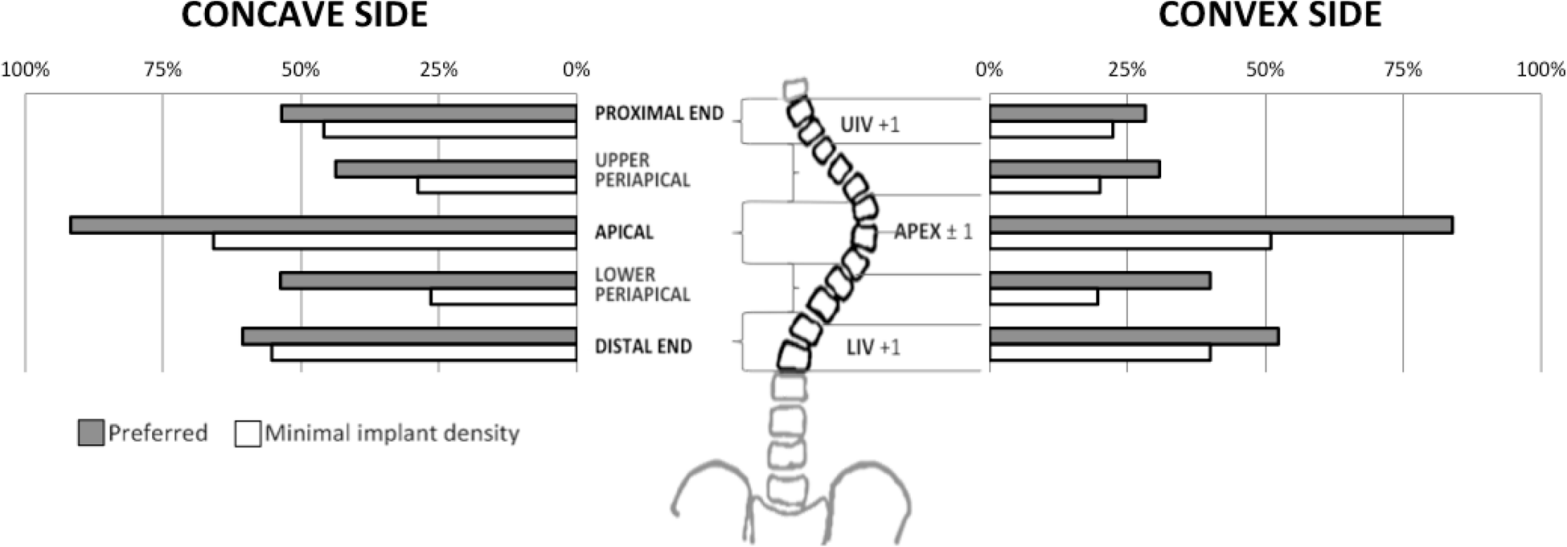
Percentage of pedicles used for correction maneuver execution on five regions for the concave and convex sides of the instrumented spine for the preferred and minimal implant density techniques

For the preferred scenario, 70% of the available implants were used to perform specific correction maneuvers, while 64% of available implants were used in the minimal density scenario (*p* < 0.05). A detailed map of anchor points necessary for the execution of compression, distraction, or derotation maneuvers was computed for each surgical plan. At each region of the instrumented spine, the mean percentage of pedicles used as anchor points for the execution of correction maneuvers was summarized (Fig. 4). The apical region had the highest rate of pedicles used for correction maneuvers (*p* < 0.01 for both scenarios) with 88% of apical pedicles were used in the preferred planning, and 58% in the minimal.

The effect of implant density on selected correction technique was then assessed. The only additional correction maneuver provided by surgeons was differential rod contouring, which does not require the use of specific anchor points. Utilization rate of each correction maneuver for the 170 surgical plans was calculated for both the preferred and minimal implant density constructs (Table 2). Lower-density constructs involved a significant decrease in the use of vertebral derotation maneuvers but an increase in the in situ rod contouring (both *p* < 0.01). Interestingly, there was no reported increased rate of Ponte osteotomies in the minimal implant density group.

**Table 2:** Utilization rate of each correction maneuver for the preferred and minimal implant density techniques

Correction maneuvers	Preferred technique (%)	Minimal technique (%)	*p* value
Ponte osteotomy	43	39	0.22
Concave rod rotation	74	80	0.13
Vertebral derotation technique	*96*	*82*	*0.003*
Segmental vertebral derotation	*54*	*39*	*0.002*
En bloc vertebral derotation	30	33	0.92
En bloc and segmental simultaneously	7	5	0.68
Compression	59	55	0.45
Distraction	71	67	0.28
In situ rod bending	*41*	*53*	*0.009*

Italic data is statistically significant (*p*>0.05)

Compression maneuvers were executed through an average of 6.69 implants for the preferred technique and 5.51 implants for the minimal implant density (*p* < 0.05). Distraction was executed through an average of 8.12 implants for the preferred technique and 6.70 implants for the minimal implant density (*p* < 0.01). For the preferred and minimal techniques, segmental derotation was executed on an average of 5.72 and 5.05 levels respectively, through an average of 1.95 and 1.76 implants per level (both *p* < 0.01), whereas en bloc maneuvers were executed on an average of 3.90 and 3.53 levels through an average of 1.73 and 1.6 implants per level (both *p* < 0.01).

**Fig. 5:**
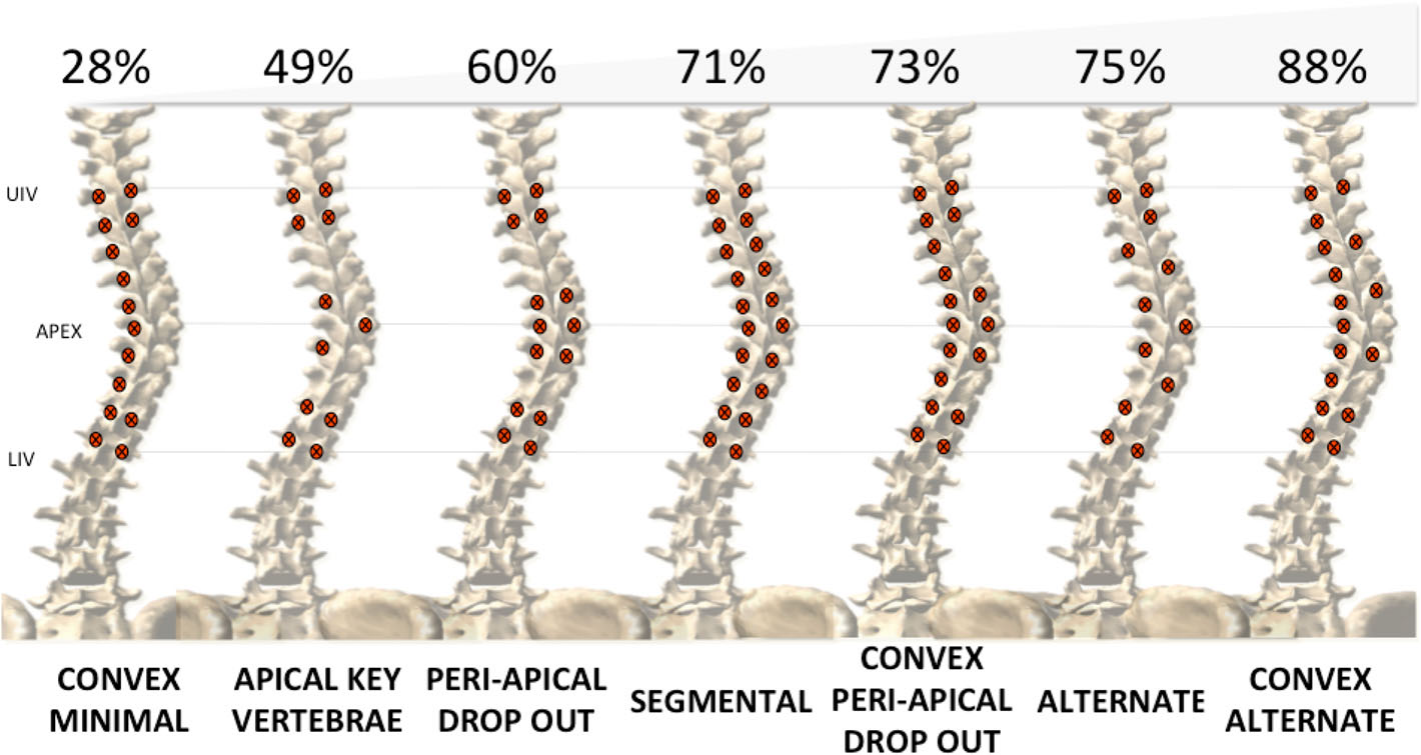
Percentage of time each implant pattern was considered to be acceptable for treatment by surgeons

Of the proposed screw patterns, level of acceptance for treatment by surgeons ranged from 28 to 88% (Fig. 5). The *convex minimal* and *apical key vertebrae* constructs were considered to be acceptable less than 50% of the time. Fleiss’ kappa ranged from *K* = − 0.06 to *K* = 0.03 which demonstrates a poor agreement among surgeons about the acceptability for treatment of all implant patterns.

## Discussion

There was significant variability in what surgeons considered an acceptable implant density for surgical planning of the same AIS cases. Some surgeons consistently used high-density constructs whereas others preferred low-density constructs. On average, surgeons selected 1.64 implants per vertebra for their preferred construct. Interestingly, De Kleuver et al. reported a consensus among international spine surgeons that less than 1.60 implant per vertebra is preferred for curves between 40 and 70° [1], which was the case for all five curve patterns in this study and less than our mean preferred construct for our surgeons. We reported that a mean of 1.25 implants per vertebra could be considered acceptable for treatment of moderate thoracic curves. Additionally in our study, for 29% of the cases, surgeons would have considered a fully instrumented construct not acceptable for treatment, which reinforces the premise that the maximal instrumentation might not represent the best option. Not every surgeon, however, was willing to alter their technique by decreasing the number of screws used.

Curve correction depends not only on implant density but also on implant distribution and the corrective maneuvers used. However, there are few studies in the literature addressing appropriate selection of implant pattern [21, 22]. With low-density constructs, a key topic is how the screws should be distributed across the spine. We found an important variability of implant distribution among surgeons, especially in the periapical region where the instrumentation rate was the lowest. Of seven screw patterns proposed in the literature, there was only a poor agreement among all surgeons about what implant pattern was acceptable or not for treatment. We reported a general acceptance among surgeons (considered acceptable in > 70% of the cases) for four of the patterns. They consisted of an alternate placement of implants on both sides or on the convex side only or skipping implants in the periapical convexity. Some patterns, such as *convex minimal* and *apical key vertebra* were less frequently selected by surgeons despite proving comparable scoliosis correction and patient satisfaction than high-density constructs [7, 19]. One explanation may be that the screw patterns were not compatible with the surgeons’ contemporary correction techniques. We analyzed the anchor points necessary for specific correction maneuver execution. While implant maneuvers were executed all along the construct, all correction maneuvers could be performed as long as there was high implant density in the apical region.

Several studies have investigated the effect of implant density on deformity correction [6]. While a debate still exists regarding appropriate implant density, many studies have compared surgical outcomes between high- and low-density constructs and provided good evidence in favor of reduced implant density [15, 17, 23, 24]. Correction techniques used for different implant density constructs were not reported, making it impossible to assess what specific changes lead to different surgical results. We investigated the effect of reducing the number of implants on reported correction techniques to better understand the role of implant density in executing correction maneuvers. Decreasing the number of implants resulted in an increased use of in situ rod bending but decreased vertebral derotation maneuvers and compression/distraction maneuvers. Such changes could significantly impact the correction, particularly in the transverse plane, which should be further investigated. It also highlights the importance of reporting and describing correction technique when comparing implant patterns as surgical results may depend not only on the number of implants used, but on the distribution of implants, stiffness of the curve, and the forces applied through specific correction maneuvers.

With implant pattern selection influencing correction techniques, it raises the question of what the optimal combination is to restore normal spinal anatomy. We noted that fewer levels and implants were used for en bloc compared to segmental derotation techniques. Hwang et al. found no difference in outcomes when comparing both techniques [25]. Although surgeons should adopt the derotation technique with which he or she is most comfortable, en bloc derotation has the advantage of requiring less instrumentation to be performed and potentially less surgical time.

The choice of implant distribution can also be guided by the capacity to share the forces during the execution of those maneuvers, and to obtain adequate post-operative stability of the construct. Biomechanical studies demonstrated that there could be downsides to using too many implants. Instead of distributing stress, numerous implants can overconstrain the construct given the limited degrees of freedom between the vertebrae and the instrumentation, thus limiting the optimal efficacy for maneuver execution and deformity correction [26]. Increased instrumentation may reduce the bony surface available for fusion. Additionally, Deriven et al. demonstrated that the addition of pedicle screws incrementally increased post-operative stability and that a simple construct with only pedicle screws placed bilaterally on the upper and lower instrumented vertebra could provide adequate post-operative stability [27].

Several limitations of this study must be considered. First, surgeons provided their surgical plans through a worksheet which may not correspond to the reality of the operating room, where the surgeons can change their preoperative planning technique depending on the progress of the surgery. Although some may state this paper represents expert opinion, rather, we consider each surgeon to be a subject who contributed surgical plans which were carefully analyzed. Secondly, the correction process is composed of a multitude of maneuvers, and by providing only the main steps of their correction techniques, surgeons may not have exactly described the sequence of maneuvers. We could not assess changes in the way surgeon execute maneuvers and evaluate whether decreasing the number of implants would lead to more force exerted on the implants. In order to facilitate comparison and consistency between cases, the selection of fused levels was set a priori by an independent surgeon. Important variability in the choice of fused levels has been previously reported [13], and correction techniques provided here might have been different if each surgeon had the liberty to select fusion levels. Similarly, we did not evaluate other factors, including rod material, rod contouring technique, and type of screws used, which can play a role in the correction process [28]. We found that implant density was similar for the five cases, but due to the limited number of cases, we were not able to fully assess if implant density and correction maneuvers correlate with curve severity or stiffness, which could be the subject of a future study. Finally, we reported an important range of instrumentation configurations that can be considered acceptable for treatment which does illustrate the lack of consensus in how to instrument routine AIS cases. Further evaluation in clinical practice is necessary to delineate what is the optimal strategy. This study draws attention to practice variability, which is a first step toward practice convergence. Further studies should investigate the cost-effectiveness of implant pattern (including important parameters such as the cost of implants and surgical duration) and their effect on health-related quality of life outcomes, as the ultimate goal of a surgical strategy is improved quality of life for patients. Despite these limitations, the design of the study allowed us to evaluate the function of implants in the correction process in order to understand the role of implant density on correction. We captured the complexity of clinical reasoning and the interdependency between instrumentation strategy and correction techniques which has to be taken into account in order to rationalize implant pattern design.

## Conclusions

Significant variability of implant density, implant distribution, and maneuvers exists among surgeons when planning surgery for the same AIS patients. Pedicle screw dropout in the convexity, particularly in the periapical regions, is globally accepted by surgeons and thought to have less potential to compromise for execution of correction maneuvers. Implant density selection resulted in adjustments to the surgeons’ correction technique. Clinical and biomechanical studies are needed to evaluate the impact of such changes, as the optimal correction technique and implant pattern have yet to be determined.

## Abbreviations

AIS: Adolescent idiopathic scoliosis
ANOVA: Analysis of variance
LIV: Lower instrumented vertebra
MIMO: Minimize Implants Maximize Outcomes
MT: Main thoracic
SD: Standard deviation
UIV: Upper instrumented vertebra
